# Critical Review of Existing MHC I Immunopeptidome Isolation Methods

**DOI:** 10.3390/molecules25225409

**Published:** 2020-11-19

**Authors:** Alexandr Kuznetsov, Alice Voronina, Vadim Govorun, Georgij Arapidi

**Affiliations:** 1 Federal Research and Clinical Center of Physical-Chemical Medicine of Federal Medical Biological Agency, 119435 Moscow, Russia; alexandr.kouznetsov.98@gmail.com (A.K.); alice_corbeau@mail.ru (A.V.); vgovorun@yandex.ru (V.G.); 2 Department of Molecular and Translational Medicine, Moscow Institute of Physics and Technology (State University), 141701 Dolgoprudny, Russia; 3 Center for Precision Genome Editing and Genetic Technologies for Biomedicine, Federal Research and Clinical Center of Physical-Chemical Medicine of Federal Medical Biological Agency, 119435 Moscow, Russia; 4 Shemyakin-Ovchinnikov Institute of Bioorganic Chemistry of the Russian Academy of Sciences, 117997 Moscow, Russia

**Keywords:** immunopeptidome, major histocompatibility complex class I, MHC, human leukocyte antigen, HLA, immunoaffinity chromatography

## Abstract

Major histocompatibility complex class I (MHC I) plays a crucial role in the development of adaptive immune response in vertebrates. MHC molecules are cell surface protein complexes loaded with short peptides and recognized by the T-cell receptors (TCR). Peptides associated with MHC are named immunopeptidome. The MHC I immunopeptidome is produced by the proteasome degradation of intracellular proteins. The knowledge of the immunopeptidome repertoire facilitates the creation of personalized antitumor or antiviral vaccines. A huge number of publications on the immunopeptidome diversity of different human and mouse biological samples—plasma, peripheral blood mononuclear cells (PBMCs), and solid tissues, including tumors—appeared in the scientific journals in the last decade. Significant immunopeptidome identification efficiency was achieved by advances in technology: the immunoprecipitation of MHC and mass spectrometry-based approaches. Researchers optimized common strategies to isolate MHC-associated peptides for individual tasks. They published many protocols with differences in the amount and type of biological sample, amount of antibodies, type and amount of insoluble support, methods of post-fractionation and purification, and approaches to LC-MS/MS identification of immunopeptidome. These parameters have a large impact on the final repertoire of isolated immunopeptidome. In this review, we summarize and compare immunopeptidome isolation techniques with an emphasis on the results obtained.

## 1. Introduction

The studies on transplantation in the 20th century led to the discovery of the antigens determining the compatibility of various tissues during transplantation [1]. These antigens were found to be presented by special transmembrane protein complexes called major histocompatibility complexes (MHCs). In humans, the products of this gene family were first found on leukocytes. Hence, the genes were called human leukocyte antigen (HLA) genes [2]. There are four groups of HLA genes (classes I, II, III, and IV), which are all located on chromosome 6. The products of these genes are proteins that differ in structure and function [3]. The HLA I and HLA II genes are among the most polymorphic human genes, and as of October 2020, 28,786 different alleles have been described for them (https://www.ebi.ac.uk/ipd/imgt/hla/stats.html). HLA I genes include the most common HLA-A, HLA-B, and HLA-C, and rare HLA-E, HLA-F, and HLA-G genes. HLA II genes incorporate HLA-DRA, HLA-DRB, HLA-DQA, HLA-DQB, HLA-DPA, HLA-DPB, HLA-DMA, HLA-DMB, HLA-DOA, and HLA-DOB [4]. The products of expression of these genes are transmembrane glycoproteins that present peptide antigens on the cell surface. The exceptions are HLA-DMA, HLA-DMB, HLA-DOA, and HLA-DOB, which regulate the loading of peptides onto MHC II molecules [5]. The main function of these molecules is to participate in the T cell-mediated immune response.

Genome-wide association studies (GWAS) have demonstrated a strong association between the presence of certain diseases and a specific HLA genotype [6,7,8,9,10]. Moreover, in several cases, the cause is HLA single nucleotide polymorphism, which affects the binding of the peptide antigen to HLA and thereby alters the repertoire of antigens presented to T cells. Table 1 lists selected studies linking HLA genotype and diseases. For example, in GWAS of 2000 Parkinson’s disease (PD) cases and 1986 healthy donors, a strong association was found between the risk of PD and the expression of the HLA-DRB5*01 and HLA-DRB1*15:01 alleles [9]. These alleles exist in about one-third of PD patients. At least one epitope obtained during the degradation of α-synuclein, which forms insoluble fibrils in filamentous inclusions of Lewy bodies in PD, is specifically presented by these forms of HLA II [11]. Similar studies made it possible to associate systemic sclerosis with the expression of HLA-DRB1*15∶02 and HLA-DRB1*16∶02 (585 cases and 458 controls) [12] and psoriasis with HLA-C*06:02 (461 psoriatic patients and 454 healthy controls) [13]. According to in silico analysis of the binding affinity of each possible fragment of the SARS-CoV-2 proteins with the expression products of 145 HLA-A, HLA-B, and HLA-C alleles, the protein product of the HLA-B*46:01 allele had the fewest predicted binding of SARS-CoV-2 peptides, which indicates a more severe course of coronavirus infection in carriers of this allele. On the contrary, the product of the HLA-B*15:03 allele is more capable of presenting highly conserved peptides of SARS-CoV-2, which improves the capabilities of T cell-based immunity [14]. Interestingly, persistent expression of a particular HLA gene can simultaneously lower the risk of developing one disease and increase the risk of developing another. For example, the instability of HLA-C makes the body more susceptible to HIV, which is why the virus seeks to suppress the expression of this gene using the Vpu protein [15,16]. On the other hand, with increased expression of HLA-C, the occurrence of Crohn’s disease [17] and psoriatic arthritis [13,18] becomes more likely. Multiple examples of this kind (Table 1), as well as larger-scale meta-analyses [19,20,21], indicate the importance of studying MHC and associated peptide antigens as a promising diagnostic tool to evaluate susceptibility to various diseases and for the development of personalized immunotherapy.

The development of mass spectrometry and peptidomic approaches to the isolation and identification of low-presented native peptides made it possible to directly determine the MHC ligands. As part of personalized cancer therapy development, mass spectrometry-based immunopeptidomics has gained the interest of biotechnological and pharmaceutical companies in the determination of peptide antigens for clinical application [34]. The goal of cancer immunotherapy is to activate the patient’s immune system and recruit their T cells, especially the CD8+ T cells, to fight the tumor. Complexes of HLA I molecules with antigenic peptides are the key to activate T killer cells. There are a significant number of oncoimmunotherapy approaches: the utilization of checkpoint blockade [35], chimeric antigen receptor (CAR) T-cell therapy [36], T-cell receptor (TCR)-engineered cells [37], T cell adoptive cell transfer (ACT) [38], and oncolytic viruses (OV)-based immunotherapy [39]. Identification of the tumor-specific immunopeptidome, as well as strategies for the isolation and genetic modification of T cells, are essential in the development of personalized cancer immunotherapy [40,41]. The diverse repertoire of HLA I presented on tumor cells is a good source of potential tumor antigens [42]. In 2018, Hilf et al. published a trial of novel personalized therapeutic vaccines (APVAC1 and APVAC2) for glioblastoma as part of the glioma actively personalized vaccine consortium (GAPVAC) [43]. The creation of these vaccines utilized published technology that includes the search for immunogenic neoantigens based on transcriptome and immunopeptidome analysis of the patient’s tumor tissue [44]. The immunogenicity of the identified peptides was verified using CD8+ T cells isolated from the patient’s blood. This highly personalized form of immunotherapy was first implemented in a global project involving a large number of research studies from various scientific centers.

In this review, we give general information about the immunopeptide and HLA, and we talk about the main methods of immunopeptidome isolation: mild acid elution and immunoaffinity chromatography. The main part of the review is devoted to various stages of immunopeptidome isolation by immunoaffinity chromatography: the choice of biological material, various detergents for the isolation of membrane-bound MHC, selection of specific antibodies, solid supports and methods for antibody immobilization, various immunopeptidome post-fractionation and purification techniques, approaches to LC-MS/MS data identification of isolated MHC ligands, and methods to confirm immunogenicity of the MHC I ligands.

## 2. General Information on Immunopeptidome and HLA

A living cell is a complex dynamic system. It has to renew its components constantly for the correct functioning. Therefore, in addition to a high-precision apparatus for protein synthesis [45], a cell requires systems to effectively remove incorrectly folded, obsolete, or unnecessary proteins. One of the main pathways for cytosolic protein degradation is the ubiquitin–proteasome system [46]. Protein candidates for degradation are labeled with the polyubiquitin protein consisting of ubiquitin monomers linked into a chain. A special complex of enzymes comprising of ubiquitin-activating enzyme (E1), ubiquitin-conjugating enzyme (E2), and ubiquitin ligase (E3) carries out the process of protein ubiquitination [46]. The proteasome, a protein machine of “creative destruction”, recognizes ubiquitinated proteins [47,48]. The proteasome contains a regulatory subunit 19S, which recognizes the substrate labeled with the polyubiquitin chain, and the proteasome nucleus 20S, cleaving the substrate. The proteasome nucleus consists of 14 different subunits, which are arranged in four folded rings with the α7β7β7α7 stoichiometry. The two outer α-rings contain seven linked identical α-subunits (α1-α7), while the inner β-rings consist of seven different β-subunits (β1–β7). Three β-subunits (β1(Y), β2(Z), and β5(MB1)) have proteolytic activities: peptidylglutamyl peptide-hydrolyzing, trypsin-like, and chymotrypsin-like, respectively [49]. At the moment, we know several types of proteasomes that have nuclei with different proteolytic properties. In addition to the classical one described above, these are immunoproteasome [50] and thymoproteasome [51]. Proteasome-mediated protein degradation results in target protein proteolysis into relatively short peptide fragments. The amino acid chain length of these fragments is regulated by endoplasmic reticulum-associated aminopeptidases (ERAP1 and ERAP2), which shorten the obtained proteolytic peptides at the N-terminus down to the size required for loading into the newly synthesized MHC I molecules [52].

**Figure 1:**
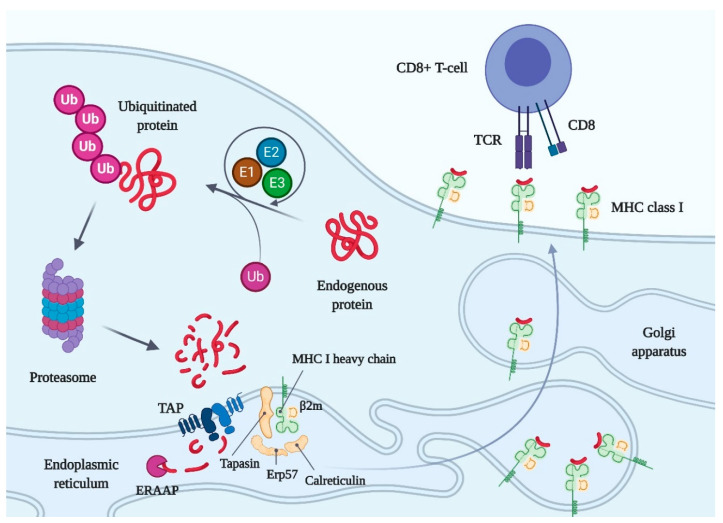
Scheme of the major histocompatibility complex class I (MHC class I) maturation, ligand binding, and presentation on the cell surface. Abbreviations: Ub, ubiquitin; E1, ubiquitin-activating enzyme; E2, ubiquitin-conjugating enzyme; E3, ubiquitin ligase; TAP, transporter associated with antigen presentation; ERAAP, endoplasmic reticulum aminopeptidase associated with antigen processing; β2m, β2 microglobulin; CD8, the cluster of differentiation 8; TCR, T-cell receptor. Created with BioRender.com.

Biosynthesis of the MHC class I molecule occurs in the endoplasmic reticulum (ER) of the cell and depends mainly on the availability of a peptide suitable for presentation (Figure 1). The synthesized MHC I heavy chain initially binds to the chaperone-like calnexin and immunoglobulin binding protein (BiP). After the non-covalent association of β2 microglobulin (light chain) with the heavy chain, calreticulin displaces calnexin. Calreticulin escorts the empty MHC I heavy chain-β2m heterodimer to a special chaperone adapter tapasin conjugated with ER-resident disulfide isomerase oxidoreductase ERp57, which forms disulfide bonds in the heavy chain of MHC I [53,54]. The lectin-like domain of calreticulin interacts with the glycan of MHC I, while its other domain (*P*-domain) provides for the interaction of the peptide-binding groove of MHC I with the ERp57 enzyme. MHC I heavy chain and β2 microglobulin, tapasin, the ERp57, lectin-like chaperone calreticulin together make up the peptide loading complex (PLC) [55,56]. Tapasin interacts with a heterodimeric peptide transporter TAP (transporter associated with antigen presentation), which delivers proteasome-cut peptides from the cytosol to the ER cavity with the consumption of ATP. Peptides transported by TAP are truncated by endoplasmic reticulum aminopeptidase associated with antigen processing (ERAAP) and loaded into an MHC I molecule, which is part of the PLC. After stabilization of the structure of the peptide–MHC I complex, the practically matured molecule, according to the classical mechanism of protein secretion, passes from the endoplasmic reticulum to the Golgi apparatus [57] and then is exposed on the cell surface as part of the vesicle (Figure 1).

Class I MHCs present peptides derived from proteins synthesized inside cells, including viral and cancer-specific proteins, on the surface of nucleated cells. The interaction of MHCs I with the T-cell receptors on CD8+ T lymphocytes mediates the detection of virus- and cancer-specific peptides and the activation of T-killer cells. Activated killer T cells can destroy antigen-presenting cells by perforins, which are similar in structure and function to the complement C9 protein [58], and granzymes [59]. Since the number of various antigens associated with danger for the homeostatic state of the body is almost infinite, the immune system must have a huge potential for distinguishing non-self. The recognition is regulated by the binding affinity of an MHC-associated antigen to a T-cell receptor. A broad repertoire of T-cell receptors [60] and a large variety of antigens associated with MHC (due to the high polymorphism of MHC within the population) are the two main mechanisms for increasing the likelihood of the appearance of a necessary MHC allele and T-cell clone in at least some individuals within the population. Therefore, it enhances the ability to fight the pathogen by adaptive immunity [61]. In the absence of MHC I on a cell surface, natural killer (NK) or NK T cells detect and kill such cells, allowing the immune system to detect the absence of the “self” marker. NK cells use special killer immunoglobulin-like receptors (KIR) to recognize MHC I molecules. The interaction of MHCs I with the T-cell receptors of immature T lymphocytes plays an important role in the positive selection of T lymphocytes in the thymus [62].

**Figure 2:**
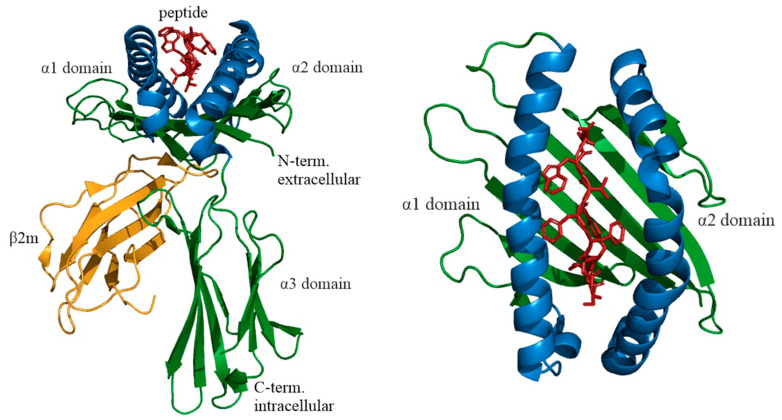
Structure of HLA-A allele group 68 (HLA-A*68) complexed with a tumor antigen derived peptide, PDB ID: 4HX1, DOI: 10.2210/pdb4HX1/pdb. Abbreviations: β2m, β2 microglobulin. Created with PyMOL.

In 1969, Mann et al. pioneered the isolation of MHC class I from mouse tissues [63]. The first detailed study of the structure of human MHC I was carried out on the product of the HLA gene allele A2 [64]. The MHC class I heavy chain consists of *N*-terminal signal peptide, which are typical for secreted proteins, three extracellular domains called α1, α2, and α3, a transmembrane domain, and a cytoplasmic domain. The light chain of MHC I is not encoded in the HLA gene. It is a small 12 kDa protein called β2 microglobulin. The α1, α2, α3 domains, and β2 microglobulin are structural homologs. The α3 domain and β2 microglobulin have a similar β-sandwich secondary fold organized into two opposing antiparallel β-sheets. α1 and α2 domains are above the α3 domain and β2 microglobulin and form a special platform consisting of eight β strands (four strands in each domain) organized into a beta-sheet and two antiparallel alpha-helices forming an antigen peptide-binding groove, which is the site of antigen binding to the MHC I molecule (Figure 2). The peptide-binding groove is around 25 Å long and 10 Å wide. The peptide-binding groove contains polymorphic amino acid residues, which allows binding to a wide range of antigens. Importantly, this peptide-binding groove of MHC I is closed at both ends. This limits the size of the presented peptide, usually of 8–12 amino acid residues, depending on the HLA I allele [65]. Certain (anchor) amino acid residues of the peptide bind to pockets of the groove. Primary anchors are usually located in the second position and *C*-terminus of the peptide, while the position of the secondary anchors is less restricted and depends on HLA I allele. For example, peptides with leucine in the second position and valine or leucine in the ninth position have a high affinity for HLA-A2. HLA-B7 typically binds peptides with proline and arginine in the second and third positions and alanine or leucine in the ninth position [66]. However, a hydrophobic C-terminal region is present in all MHC I peptides [65]. MHC I molecules can also present longer peptides (up to 25 aa) due to the protrusion of weak affinity regions of the peptide chain and preservation of the positions of anchor amino acid residues [67].

## 3. Methods for Immunopeptidome Analysis

An important milestone in the studies of the immunopeptidome of various animal cells was a creation of the method for the isolation of MHC I ligands by mild acid elution (MAE) proposed by Sugawara et al. in 1987 [68]. The essence of this easy-to-implement method is the short-term treatment of living cells with citrate buffer (pH 3.0). As a result of such treatment, the β2 microglobulin molecule non-covalently bound to the MHC I heavy chain dissociates, destabilizing the structure of the entire complex. This reduces the peptide-binding capacity of the HLA-A, HLA-B, and HLA-C complexes, i.e., it leads to the loss of peptides associated with the MHC class I molecules [68]. The hypothesis was made that MHC class II molecules do not lose their antigens during MAE, which increases the specificity of the technique. The assumption was confirmed a little later [69]. Importantly, working with cells by the MAE method leaves them viable with the ability to regenerate MHC I complexes with antigens, which facilitates the accumulation of a significant amount of MHC I ligands. At the time MAE was proposed, which allowed using no more than 100 million cells, it was indeed an extremely effective technique compared to other methods used for the isolation of the MHC I peptidome (trifluoroacetic acid extraction [70] and immunoaffinity isolation using specific antibodies [65]), requiring 1–10 billion cells. The growing interest in immunopeptidomics and a significant amount of accumulated experimental data have stimulated the emergence of several detailed reviews and comparative works related to the MAE method [71,72,73,74,75]. Undoubtedly, the simplicity and efficiency of MAE [68], including a small number of purification steps, the absence of detergents [72], the possibility of multiple processing of living cells [76], and the reduction of losses in the case of working with low-affinity peptides [72] made the MAE method one of the main tools of immunopeptidomics. On the other hand, the need to work with living cells is one of the most significant weaknesses of the MAE method, which is highlighted by many researchers. In addition, elution should take place in a cell suspension; that is, cells should circulate freely in solution [73]. Hence, it is not possible to use MAE on tissues and cell lines requiring special conditions for growth. Even more problematic is the simultaneous elution of peptides present in large amounts on the cell surface and not related to the MHC I ligandome. According to Fortier et al., only about 40% of all peptides isolated by the MAE method are associated with MHC class I, while the rest are contaminants [68,72,77].

**Figure 3:**
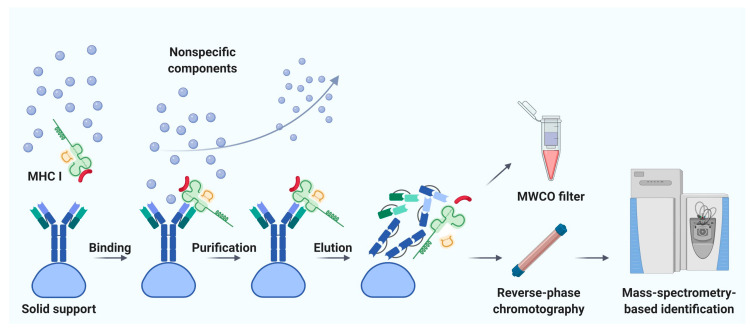
Immunoaffinity chromatography. Abbreviations: MHC I, major histocompatibility complex class I; MWCO, molecular weight cutoff. Created with BioRender.com.

Immunoaffinity chromatography is a method for the isolation and purification of a target substance from a multicomponent mixture based on a specific non-covalent interaction of an antibody immobilized on a solid support and an antigenic epitope of the target substance [78]. Unlike MAE, immunoaffinity chromatography finds applications in various fields of biomedicine, including clinical diagnostics, detection of substances hazardous to the environment, and pharmacological research [79]. The basic principle of immunity chromatography is still the same, despite the constant improvement of methodology [74,80,81,82]. A multicomponent mixture featuring a cell line lysate, homogenized tissue, or biological fluid sample is incubated with MHC-specific antibodies pre-immobilized on magnetic particles or agarose-based polymeric resins as solid support (Figure 3) [79]. The murine monoclonal antibody, clone W6/32, which specifically binds to the α2–α3 heavy chain region of the products of all classical genes HLA-A, HLA-B, and HLA-C is commonly used [74,82]. After purification from non-specifically bound substances, MHC molecules together with associated peptides are eluted. Currently, the method of immunoaffinity purification is the most commonly used for isolating an immunopeptidome. There are reasons for this: (1) most of the peptides isolated by this method can be true ligands of MHC; several studies bioinformatically confirm the high affinity for MHC in about 90% of identifications [83,84,85], and (2) this method is less demanding on the biomaterial; it is possible to use both cell lines and tissues, biological fluids, including frozen samples.

Noteworthy, the labor and time costs of this method are higher compared to MAE. Immunoaffinity chromatography for the isolation of MHC requires a significant amount of specific antibodies; therefore, there is a need to maintain an in-house hybridoma producing the required antibodies [86,87]. On average, about 1 mg of antibodies per sample is required [88]. It is not surprising that, to our knowledge, the largest published work to date is devoted to the study of the immunopeptidome of only 10 biological samples of postoperative material and 142 samples of blood plasma [89]. Using isotopically labeled peptides, Hassan and co-authors found that losses during immunoprecipitation of the MHC ligandome reached 90–99% [90]. Due to the large number of washes required to get rid of non-specific peptides, there is a high risk of losing low-affinity MHC ligands [71]. In addition, it is still not precisely established how universal the antibodies are—that is, whether there are such MHC variants that bind antibodies with low affinity and, as a result, some of the MHC-ligand complexes are lost [91]. Taking into account all sources of loss, it is not surprising that the number of cells required for successful LC-MS/MS identification of the MHC ligandome varies from 100 million to 10 billion [92]. However, attempts are being made to improve methods of immunoaffinity purification [93]. Chong et al. propose to accelerate and automate the protocol by carrying out immunoprecipitation in 96-well plates. The researchers isolated 42,556 unique MHC class I associated peptides belonging to 8975 precursor proteins, using 21 wells containing 100 million cells each [93]. Out of 10 million cells, they managed to identify only 1846 peptides, but these 1846 peptides are almost the same as the most represented peptides isolated from 100 million cells. Lanoix and co-authors published a comparison of the quality of the B-cell lymphoblast immunopeptidome isolation by MAE and immunoprecipitation [73]. As a result of the isolation of immunopeptidome from 2, 20, and 100 million cells, the authors managed to identify 2016, 3931, and 5093 unique peptides by immunoaffinity chromatography and 314, 2081, and 2996 unique peptides by MAE with MS detection. Thus, more peptides associated with HLA I were obtained by immunoaffinity purification. However, the difference in the total amount of isolated peptides with an increase in the initial number of cells aligns between the two methods.

It is the isolation of the immunopeptidome that some authors aptly call an Achilles’ heel, hinting at an inhibitory effect on the development of the research area as a whole [88]. Indeed, back in 1992, Hunt et al. showed that the majority of peptides presented via MHC I varies from 100 to 1000 copies per cell, and only a few are present in 1000 to 3000 molecules per cell [80]. In some cases, the representation of a single peptide can reach 10,000 copies per cell [94]. Moreover, according to the data of Schuster et al., the average number of HLA I molecules per cell varies from 5000 to 150,000 [95], and according to Lanoix et al., the total number of MHC I per cell can reach 0.5–3 million [73], which theoretically allows the cell to present 10,000–30,000 different peptides. If we take into account that losses during immunoprecipitation of the MHC I ligandome can reach 90–99% [90], we can isolate 1 to 300 million molecules of each peptide from 1 million cells, which approximately corresponds to amounts from 2 amol to 0.5 fmol. As the limiting sensitivity of LC-MS/MS, one can take the result obtained by Matthias Mann’s group in 2010 on Orbitrap Exactive [96]. Using the Universal Proteomics Standard (UPS1), they identified 348 different peptides, in triplicate, from 45 of 48 UPS1 proteins using the 140 fmol of corresponding tryptic peptides. Although the identification was performed against a database of all human proteins, the sensitivity would be lower under conditions of a high dynamic range of real biological samples. If we take 500 fmol of a peptide as a sufficient amount, then for successful identification of the peptide in the immunopeptidome, at least 1 billion cells should be taken, which is roughly consistent with the scale of current works on immunoprecipitation [88,90,92,95].

The study on the regulation of the presentation of the HLA I peptide repertoire is an important task [50,97,98,99]. The detection of factors capable of increasing the amount of MHC presented by a cell can reduce the required volume of biological material and/or increase the number of different detectable MHC ligands. Javitt and co-authors show that pro-inflammatory cytokines tumor necrosis factor alpha (TNFα) and interferon gamma (IFNγ) increase the number of identifiable HLA I ligands in the lung epithelial cell line A549 from 3444 unique peptides without cytokine treatment to 6582 unique peptides after the treatment [99]. About 500 million cells were used in a single experiment. The authors showed that the pro-inflammatory molecules TNFα and INFγ increased the diversity of immunopeptidome, which was due to the functioning of a special immunoproteasome synthesized in cells under the effect of these cytokines [49].

Another method for isolation of HLA I molecules and their ligandome is the transfection of a cell line with an expression vector encoding a soluble secreted form of MHC I, without a transmembrane domain, and the further immunoprecipitation of secreted MHCs with peptides attached. The MHC I delivery methods include DNA transfection [100,101], transduction with retroviruses [102], and mRNA transfection [103]. At the same time, this method allows culturing cells for long periods, similar to MAE, which facilitates the accumulation of a significant amount of MHC ligands and gives the most specific result due to the immunoprecipitation. However, various genetic engineering procedures can cause an appreciable rearrangement of the protein composition of the cell, together with the MHC ligandome. In addition, similar to MAE, this method does not work with tissues due to the complexity of the use of genetic engineering techniques [74].

## 4. Current Protocols for Affinity Chromatography

In the last decade, there has been an explosive increase in the number of publications on the immunopeptidome of various biological samples. Researchers optimized immunoaffinity chromatography to isolate MHC-associated peptides for individual tasks and published a mammoth amount of protocols with differences in parameters that have an impact on the final repertoire of isolated immunopeptidome. We have divided this chapter according to the stages of immunopeptidome research based on immunoaffinity chromatography: the choice of biological material, various detergents for the isolation of membrane-bound MHC, the selection of specific antibodies, solid supports and methods for antibody immobilization, various immunopeptidome post-fractionation and purification techniques, approaches to LC-MS/MS identification of isolated MHC ligands, and methods to confirm immunogenicity of the MHC I ligands. We quote the most interesting publications from our point of view, and everyone interested in a particular stage of immunoprecipitation could, using the examples of these works, evaluate the effectiveness of the approach and look at them for methodological recommendations.

### 4.1. The Choice of Biological Material

An important step in planning an immunoprecipitation experiment is the selection of the biomaterial. The sources of MHC I molecules are quite diverse: cell lines, tissue biopsies (including tumors), blood (both peripheral blood mononuclear cells (PBMCs), and soluble in plasma forms of MHC), or other biological fluids. Solid tissue is best suited for finding MHC-associated peptide biomarkers or targets for therapy. However, for subsequent routine screening, a repeated biopsy is not acceptable. On the other hand, blood is the most common connective tissue in the body, containing a large number of metabolic products produced by various organs and tissues [104]. In addition, blood is a biomaterial used routinely in diagnostics. In addition to cell membranal HLA (mHLA) molecules, soluble HLA (sHLA) molecules circulate freely in the bloodstream [105,106]. The soluble forms of HLA are either a result of the cleavage of various metalloproteinases of membrane HLA [107] or products of alternatively spliced HLA genes [108]. Normally, there is a low concentration of sHLA molecules in the bloodstream. However, many types of tumor cells release a significant amount of sHLA, and this phenomenon can be used in cancer diagnostics [105]. In the first large-scale studies of sHLA, Bassani-Sternberg and co-authors identified more than 12,000 unique peptides using small blood samples of 2–5 mL from 12 donors [106]. Since the HLA proteolytic site for cleavage by metalloproteases can also be utilized by papain [107], in several studies, the authors treated cells with papain to produce sHLA [109], purified them by immunoprecipitation, and identified HLA I ligands [110].

The study of tumor HLA-associated antigens in glioblastoma multiforme (GBM) is an example of a compromise in the use of biomaterial [89]. The main data on sHLA were obtained from 142 blood plasma samples, whereas for correlation with tumor-associated mHLA, postoperative biopsies were analyzed from only 10 patients. There was a strong correlation in the peptide patterns of tumor tissues and blood samples from the same donor. Based on 35,545 identified peptides, the authors found potential biomarkers specific for GBM. Interestingly, their concentration in the bloodstream significantly decreased after tumor removal. For sHLA analysis, the authors used 2.5–15 mL of blood plasma, allowing them to identify, on average, 1774 peptides 8–14 aa long from a sample. In mHLA analysis using 0.3–0.5 g of the tumor tissue, they identified, on average, 4359 peptides 8–14 aa long from a sample.

In the study on the identification of MHC-presented antigens of Mycobacterium tuberculosis for the development of a new vaccine, Bettencourt et al. worked on the THP-1 cell line of macrophages infected with the bacillus Calmette-Guérin (BCG) vaccine [111]. In a set of experiments, the authors used from 50 to 500 million cells per sample, which allowed them to identify a total of 23,976 unique MHC I associated peptides, of which 43 peptides derived from 41 M. tuberculosis antigens.

For the detection of peptide vaccine targets, Berlin and co-authors analyzed PBMCs from acute myeloid leukemia (AML) patients [112]. They isolated 0.2–19 billion cells from the patients’ blood and, using flow cytometry, they found that AML blasts contained from 45,189 to 261,647 HLA I molecules per cell. LC-MS/MS analysis of an HLA I immunopeptidome from 15 AML patients identified 13,238 HLA I ligands related to 6104 proteins, from which they formed a panel of 132 ligandome-derived tumor-associated antigens (LiTAAs).

### 4.2. Various Detergents for the Isolation of Membrane-Bound MHC

One of the cornerstones during the work with immunopeptidome is the isolation of membrane-bound MHC I molecules from biological material as cells or tissue so as not to lose MHC associated peptides. Therefore, it is necessary to pay special attention to the applied method of lysis, namely the detergent used. Detergents are amphipathic molecules that contain both polar (e.g., phosphoric acid residue or carboxyl group) and non-polar groups (e.g., long aliphatic chain). Detergents are capable of forming aggregate micelles and a waterless space with the non-polar parts, while the polar ones contact the surface of water. At low concentration of the detergent, its molecules can incorporate into a cell bilipid membrane layer. With an increase of the detergent concentration, the destruction of the bilipid membrane layer occurs with the formation of micelles, which include membrane lipids, membrane proteins, and the detergent molecules. All detergents can be classified based on the properties of the polar part: ionic (anionic, cationic), non-ionic, and zwitterionic [113]. Among the ionic detergents used when working with MHC I, the most commonly applied are negatively charged sodium salt of deoxycholic acid [89,114]. Speaking about non-ionic detergents used for the isolation of MHC I, it is necessary to mention Triton X-100 (Triton) [115], NP40 [80], IGEPAL CA-630 (Igepal) [111,116], and *N*-octyl-β-d-glucopyranoside [84,99]. Concerning zwitterionic detergents, the most common is 3-[(3-cholamidopropyl) dimethylammonio]-1-propanesulfonate (CHAPS) [73,112,117,118]. Detergents of different groups can be used together, such as *N*-octyl-β-d-glucopyranoside and sodium deoxycholate [84,89,99,114]. Nicastri et al. immunoprecipitated MHC I from Jurkat cell line (300 million cells per sample) with four different detergents: Igepal, Triton X-100, CHAPS, and sodium deoxycholate [119]. Each detergent, except for sodium deoxycholate, was taken in two concentrations: half or twice the reported micelle forming concentration. According to their results, the peptide yield was higher for samples lysed with higher concentrations of detergents. Among detergents taken in twice the reported micelle forming concentration, most peptides were identified in samples lysed with CHAPS, followed by IGEPAL, Triton, and sodium deoxycholate: 4420, 4205, 3750, and 3617 peptide sequences identified on average, respectively. The authors also studied the differences in identified peptides for the main HLA I alleles of the Jurkat cell line with the help of NetMHCpan 4.0 depending on the detergent used. Lysis with CHAPS 0.74% helped identify more peptides assigned to HLA-B*07:02 and HLA-B*35:03 alleles. Triton 0.1% provides more HLA-A*03:01-related peptides. The highest number of peptides not predicted to bind the main HLA I alleles of Jurkat cell line was detected in samples processed with sodium deoxycholate 0.25% and IGEPAL 0.1%.

Partridge et al. demonstrated that some of the peptides identified as a result of the standard HLA I ligands immunoprecipitation protocol (lysis buffer with IGEPAL) are not associated with HLA I [120]. The authors showed that HLA II bound peptides, as well as membrane proteins of HIV-1-infected cells, can be co-purified on antibodies specific to HLA I. Researchers suggested that cell membranes were only partially destroyed during the lysis, which led to the formation of very small membrane fragments, with several non-target proteins were incorporated into them besides MHC I. Thus, lysis protocols for immunoprecipitation still have some ambiguities that require further development.

### 4.3. Selection of Specific Antibodies

The advantage of immunoprecipitation-based MHC studies is their high specificity and accurate analysis of individual types of the extremely diverse MHC repertoire, using antibodies specific for a particular MHC allele. For example, for the HLA-B*27 allele associated with severe autoimmune disease, ankylosing spondylitis, Sanz-Bravo et al. used monoclonal antibody ME1 (IgG1) against the HLA-B*07/B*27/B*22 alleles. This antibody recognizes the whole complex of the HLA heavy chain, β2 microglobulin, and the peptide [121]. By selecting antibodies for research tasks, one can achieve more accurate and detailed results. The list of antibodies commonly used to isolate HLA can be found in various protocols [74,82,122]. The most commonly used antibody for HLA I isolation is mouse monoclonal antibody W6/32, which is stereospecific to the heavy chain of HLA-A, HLA-B, and HLA-C molecules, binding the α2-α3 region [123]. Monomorphic antibodies, such as W6/32, recognize monomorphic determinants that are common to all HLA class I alleles, whereas polymorphic antibodies (ME1 against HLA-B*07, BB7.2 against HLA-A*02, GAP.A3 against HLA-A*03, etc.) recognize determinants carried by specific allele [124].

The other antibody that is commonly used for the immunoprecipitation of HLA I is BB7.2, which has an allotype specificity against HLA-A*02 and HLA-A*69 [124]. In one of the first classical characterizations of the specific HLA I allotype immunopeptidome, Hunt et al. used BB7.2 antibody to purify HLA-A*02 from the human B lymphoblastoid cell line C1R-A2.1 (2 billion cells per sample) [80]. This cell line was HLA-A*02 transfectant of the HMy2.C1R cell line, which did not express HLA-A and HLA-B. The total number of identified peptides was 200. Almost 30 years later, Pandey et al. identified practically a hundred times more (20,316 HLA-A*02:01 ligands) from 1 billion HMy2.C1R cells transfected with HLA-A*02:01 [125]. The transfection of MHC I-deficient cells with a certain MHC I allele greatly facilitates the identification of MHC I-associated peptides since it removes the ambiguity that arises from the co-expression of multiple MHC I alleles. In this way, one can investigate the eluted peptides that came from the allele of interest [115]. There are several so-called “HLA-null” cell lines, which do not express any HLA I alleles and can be used for these purposes, the most common of which are human immortalized myelogenous leukemia cell line K562 [126] and the human B lymphoblastoid cell line 721.221 [115,127]. Concerning 721.221 cell line, Partridge et al. in 2018 showed that it actually expresses and presents peptides on HLA-C*01:02 [120].

Since immunoaffinity purification requires a significant amount of specific antibodies (about 1 mg of antibodies per 1 g of tissue or 1 billion cells [74]), the most suitable method for obtaining these antibodies is to use the desired hybridoma. In the research of Pandey et al., authors used HB-82 hybridoma for producing BB7.2 antibodies. Bassani-Sternberg and co-authors used the HB-95 hybridoma as a source of the W6/32 antibodies to study how the representation of a protein peptide fragments in complexes with HLA I depends on the amount of this protein in a cell in a collection of seven tumor cell lines. The authors identified 22,244 unique peptide sequences, of which 93% were 9-11 amino acid residues long [84].

### 4.4. Solid Supports and Methods for Antibody Immobilization

Immunoaffinity chromatography uses a solid matrix with immobilized antibodies for the convenience of further manipulations. One of the most common carriers is sepharose, which is cross-linked agarose granulated in a special way. One usually puts it into solution and sediment at each washing step by centrifugation or uses it as a resin to fill a chromatography column. Schuster et al. used a sepharose resin with immobilized HLA I-specific monoclonal antibodies W6/32 to examine the epithelial ovarian cancer (EOC) immunopeptidome. In 42 tissue samples (0.5–3 g of tissue or 0.25–1 billion cells per sample), they were able to identify 34,177 unique peptides, which were fragments of 10,677 proteins [95]. In a comparative analysis with the data of HLA I ligands in benign tumors (liver, colon, ovary, and kidney), as well as in PBMCs from healthy donors, 1143 peptides were found to be specific for EOC tissues. Among them, 113 peptides from mucin 16 (MUC16) protein, one of the constantly expressed EOC markers, were identified.

In addition to polysaccharide beads, magnetic beads are often used as solid support. The presence of a metal center in the particle allows simplifying and speeding up work with them through using a special magnetic stand. Moreover, the use of modern robotic platforms makes it possible to automate the process. However, sepharose columns allow working with several types of antibodies simultaneously, for example, to independently isolate MHC I and MHC II and push a large volume of biomaterial through such a column in cycles [74]. Lanoix and co-authors isolated the immunopeptidome of the Epstein–Barr virus-transformed B-lymphoblastoid cell line by immunoprecipitation on magnetic particles [73]. When isolating from 2, 20, and 100 million cells, they managed to identify 2016, 3931, and 5093 peptides, respectively.

Researchers immobilize specific antibodies on magnetic or sepharose particles by covalent and non-covalent attachment. We will present only a few of the most popular techniques used in works with HLA I molecules and associated peptides. One of the main ways to bind specific antibodies to a solid support is covalent attachment [74,82]. Reactive groups must be created on the surface of the carrier particles to react with the antibody. Most often, they are electrophilic groups that are capable of interacting with the nucleophilic groups of the ligand, for example, with the ε-amino group of lysine, sulfhydryl group of cysteine, hydroxyl group of tyrosine, and terminal amino group [128]. In this case, the process of ligand immobilization comes down to its incubation with an activated matrix. There are many commercially available options for support activation. However, in this review, we focus on two of them: activation with cyanogen bromide (CNBr) and with aldehydes. CNBr binds to the hydroxyl groups of sepharose to form imidocarbonate. The electrophilic carbon atom in this compound reacts with the nucleophilic side groups of amino acids [128]. The advantage of this method is its simplicity, high stability of formed bonds, and sepharose stability in a wide range of pH from 2.0 to 12.0. However, this method has a significant disadvantage. The by-product of the reaction–isourea derivative causes non-specific binding. On the other hand, many immunopeptidome studies use the cyanogen bromide activation method and show a large number of identified HLA ligands. For instance, in the above-mentioned works by Schuster et al. and Berlin et al., the authors were able to identify 1334 and 1299 unique peptides per sample from 0.5–3 g EOC tissue and 0.2–19 billion PBMCs, respectively [95,112].

Reductive amination of aldehydes is another way of protein covalent binding to the resin, which does not cause non-specific binding. The aldehyde group on the support reacts with the amino group of the antibody to form a Schiff base, which is then reduced by a mild reducing agent, cyanoborohydride [128]. This reaction can be performed at normal pH 7.2; however, carrying it out under more basic conditions pH 9–10 promotes the formation of Schiff bases. For the study of immunopeptidome of five human cancer cell lines: multiple myeloma RPMI8226, acute myeloid leukemia HL-60, acute monocytic leukemia THP-1, embryonal kidney HEK293, and mantle cell lymphoma MAVER-1 cells, Ritz and co-authors used aldehyde-activated agarose beads for covalent coupling of antibodies [85]. Researchers analyzed 100 million cells per cell line and identified 4932, 7155, 5999, 5389, and 7865 unique peptides for lines RPMI8226, HL-60, THP-1, HEK293, and MAVER-1, respectively. Using the same experimental approach, the authors managed to identify 306 to 972 unique sHLA-associated peptides from 4–5 mL of blood serum of eight melanoma patients and four healthy donors. The previously mentioned work by Shraibman et al. on the identification of the glioblastoma immunopeptidome also used an aldehyde-activated agarose resin for antibody immobilization [89]. From 2.5 to 15 mL of blood plasma and 0.3 to 0.5 g of tumor tissue, they identified on average 1774 and 4359 peptides, correspondingly.

Among the methods of non-covalent attachment of antibodies to the support for the isolation of HLA I and its ligands, the most popular are bacterial proteins A first found in the cell wall of Staphylococcus aureus and protein G first isolated from the cell wall of Group G Streptococci, which bind Fc fragments of antibodies [129]. Protein A and protein G have different binding capacities for different types of antibodies. However, a recombinant protein consisting of four Fc-binding domains of protein A and two Fc-binding domains of protein G solves this problem. To identify neoantigens of native human melanoma, Bassani-Sternberg and co-authors used agarose resin with protein A [114]. In addition, the authors used a dimethyl pimelimidate crosslinker to covalently bind antibodies to protein A through the corresponding primary amino groups. The authors identified 78,605 HLA I peptides from 12,663 proteins in biopsy samples from 25 patients (the amount of biomaterial taken varied from 0.1 to 4 g), including 64 fragments of a well-known melanoma-associated antigen PMEL.

### 4.5. Various Immunopeptidome Post-Fractionation and Purification Techniques

Before the mass spectrometric step, the isolated MHC ligands require additional purification from salts, interfering with the ionization of the analyzed peptides in a mass spectrometer. The most common desalting method is solid-phase extraction (SPE). Peptides and proteins that are more hydrophobic bind to a sorbent, most often with C18 hydrophobic groups, while water-soluble hydrophilic components, including salts, are washed out. Quite often after immunoaffinity purification, the resulting eluates are additionally purified from proteins (antibody fragments, MHC heavy chain, and β2 microglobulin) by centrifugation through 3–10 kDa molecular weight cutoff (MWCO) filters. In their protocol, Nelda et al. advise first to separate the MHC peptides from proteins: 3 kDa filter units for short MHC class I ligands 8–12 amino acid residues long; 10 kDa filter units for longer MHC class II ligands 12–25 amino acid residues long, and then desalt the samples with ZipTip C18 pipette tips [74]. However, each additional purification step leads to the loss of the target product, whereas peptides can be separated from high molecular-weight proteins using the same reverse phase chromatography. Ritz and co-authors evaluated the necessity to use MWCO filters, replacing them by stepwise elution from C18 resin [85]. Using elution with 30 and 80% acetonitrile, they were able to separate the peptide and the HLA fractions, respectively. The simpler protocol, without the use of MWCO filters, yielded a higher number of identified peptides and was used in the main part of the work. The authors identified 27,862 unique peptides in five human cancer cell lines. Likewise, in a study of low-abundant peptides of human papillomavirus, Blatnik et al. optimized the protocol for the isolation of HLA I ligands and achieved an increase in the number of identified peptides by abandoning the ultrafiltration step and leaving only the reverse phase chromatography [116].

The almost ubiquitous application of reversed-phase chromatography for desalting creates a bias in the detectable immunopeptidome due to its high biochemical diversity and low representation of each peptide [130]. Comparing two variants of HLA I ligandome fractionation (high-pH reversed-phase and strong cation exchange), Demmers and co-authors concluded that although fractionation allows identifying more peptides, it can lead to HLA I allele-specific ligand identification bias. In addition, hydrophobic peptides can be lost due to irreversible binding to conventional reversed-phase C18. Rappsilber et al. created a protocol with the use of homemade stop-and-go-extraction tips (StageTips). Those are pipette tips with very small disks made of beads embedded in a Teflon mesh [131]. Many materials with particles of different adsorption properties: with reversed-phase, cation-exchange, or anion-exchange surfaces, or even titania and zirconia are suitable for this protocol, enabling the researchers to select a combination of sorbents for each particular research task. Demonstrating properties of various sorbents, Kulak and co-authors showed in the proteome experiments that StageTips with poly(styrenedivinylbenzene) reverse-phase sulfonate (SDB-RPS) allows the isolation of about 20% more tryptic peptides than StageTips with conventional reversed-phase C18 [132], since SDB-RPS has a greater affinity for more hydrophobic peptides. We assume that the use of this technique and appropriate sorbents can increase the final yield in the isolation of the immunopeptidome.

Noteworthy, peptides presented by MHC can carry biologically important post-translational modifications [133]. Therefore, methods for sample enrichment with modified peptides, including phosphorylated and glycosylated ones, have been developed. One of the most convenient methods to work with phosphopeptides is immobilized-metal affinity chromatography (IMAC). The reversible pH-dependent interaction between phosphorylated amino acids acting as electron donors and metal ions attached to a solid resin (for example, Fe^3+^ or Ti^4+^) allows enrichment of the sample [134]. Abelin et al. published a protocol for Fe^3+^ IMAC enrichment of phosphorylated HLA I ligands from cell lines and tissue samples [118]. Using 100 million to 1 billion cells or equivalent amounts of tissue, they increased the number of phosphopeptides to over 95% of the total number of identified peptides. Before the enrichment, phosphorylated peptides comprised 1–5% of the immunopeptidome. From 0.6 g of colorectal cancer tissue, using two types of Fe^3+^ IMAC, they identified 84 phosphorylated peptides. To enrich immunopeptidome with glycosylated peptides, lectin-agarose affinity columns [135] or amino-phenyl boronic acid (APBA) derivatized POROS beads [136] can be used. Malaker and co-authors isolated and identified 36 MHC I ligands from 0.1–1 billion cells using *O*-linked β-*N*-acetylglucosamine. Notably, five of the seven analyzed glycopeptides were found immunogenic in the ELISpot IFNγ assay [137].

### 4.6. Approaches to LC-MS/MS Data Identification of Isolated MHC Ligands

High-performance Orbitrap (Thermo Fisher Scientific) and TripleTOF (SCIEX) mass spectrometers are the most suitable mass spectrometers for the simultaneous analysis of many thousands of endogenous peptides presented via MHC [137]. The overwhelming majority of shotgun immunopeptidome studies are carried out on various modifications of Orbitrap [73,84,85,89,95,99,106,111,112,114,118,136]. Reversed-phase high-performance liquid chromatography (RP-HPLC) precedes mass spectrometric analysis for greater efficiency. As a rule, the collision-induced dissociation (CID) fragmentation is used, but there are examples of the application of hybrid fragmentation methods [83]. Mass spectrometric approaches and bioinformatic methods of immunopeptidome analysis are largely taken from the field of shotgun proteomics, but there are some fundamental differences. First of all, in the analysis of endogenous peptides, the stage of proteolysis, which introduces bias into the representation of proteins in proteomic approaches, is completely absent [138,139]. On the other hand, the identifications of MHC peptide ligands are independent, while tryptic peptides are combined into precursor proteins at the stage of bioinformatic analysis, which increases the reliability of each peptide identification.

Unlike tryptic peptides, endogenous peptides of various MHC alleles do not have a characteristic pattern of hydrolysis, and there may be no lysine or arginine at the C-terminus of such peptides, which increases the signal of the so-called y-ion series. As a result, the immunopeptidome has a less distinct spectrum of fragmentation and, therefore, a lower chance of identification. Hybrid fragmentation methods are used to improve the quality of the fragmentation spectra of MHC ligands [83]. Momme et al. significantly increased the number of identified peptides by analyzing a combination of collision-induced dissociation (CID), beam-type higher-energy CID (HCD), and electron-transfer/higher-energy collision dissociation (EThcD) spectra. After analyzing the LC-MS/MS data obtained for the GR B-lymphoblastoid cell line immunopeptidome using CID/HCD, EThcD, and combined methods, the authors managed to identify 9015, 6381, and 12,199 unique peptides, respectively. Ritz and co-authors analyzed the immunopeptidome of five cell lines by LC-MS/MS on a Q Exactive mass spectrometer and used two search algorithms to increase the number of identified peptides [85]. Application of the Proteome Discoverer and MaxQuant allowed them to identify from 4932 to 7865 unique peptides for different cell lines, and the unique contribution of Proteome Discoverer and MaxQuant being about 28 and 19%, respectively. Thus, they were able to improve the analysis result by about 20% just by adding an alternative search tool. Among search algorithms, MSFragger is now gaining more and more popularity, since it allows comprehensive peptide identification (without specifying the protease specificity, including post-translational modifications (PTMs) in a fairly reasonable time [140].

Unlike tryptic peptides, MHC ligands have low confidence of peptide spectra matching scores, mainly due to a lack of enzyme restriction in the searches. At the stage of identification of mass spectra, the search space significantly increases, which leads not only to an increase in the analysis time but also to an increase in the number of false-positive identifications in comparison with a similar analysis of tryptic peptides. In addition, the search space increases further if we analyze the possible splice variants of peptides formed by the proteasome [141,142], peptide modifications [118,135,136], and genomic mutations [143]. To partially neutralize the problem of low confidence of peptide spectra matching, machine learning and artificial intelligence approaches are actively used. For effective training of these algorithms, databases of mass spectrometric data and spectral libraries of immunopeptidomes, such as, for example, SysteMHC [144], are created. A targeted database search of MS data was proposed by Murphy et al.: the corresponding SpectMHC tool predicts possible ligands for the analyzed MHC alleles and then identifies peptides against the predicted peptide sequence database [145]. Allele-specific peptide databases for mice and humans were created. Identification using these databases showed more than twice as many potential MHC ligands than the traditional search with no enzyme specificity. Liepe et al. also included proteasomal cis-spliced peptides in the database of potential MHC ligands [146]. Using only Mascot as a search algorithm, they were able to show that in breast and colorectal cancer cell lines, spliced peptides make up about 20% of the MHC I immunopeptidome. Despite the fact that, according to another estimate, spliced peptides represent only 1–2% of the total amount of peptides formed by proteasome-mediated degradation, it is believed that spliced peptides are importantly involved in some processes of oncosuppression [147]. Another possible solution is a combination of approaches for identification against a protein database and de novo sequencing algorithms, which are offered by the developers of recently the most popular de novo analysis software PEAKS [148]. Improvements in the resolution and accuracy of mass determination of modern mass spectrometers led to the increasing popularity of de novo sequencing algorithms, since they do not require a priori knowledge of all analyzed protein sequences [149].

### 4.7. Methods to Confirm Immunogenicity of the MHC I Ligands

Although most authors in the field use the term immunopeptidome to describe the set of all peptides presented by MHC, in our opinion, the more correct term, in this case, is MHC ligandome [150]. Strictly speaking, an immunopeptidome is the collection of all peptides involved in the biochemistry of the immune system. The mass spectrometry-based peptidomics approaches used today for high-throughput study of MHC ligands by themselves do not allow the detection of immunological activity for all identified peptides (at least to confirm the interaction with the T-cell receptor). Not all peptides presented as part of HLA I complexes on the surface of cells have immunogenic properties; that is, they can activate T cells and thereby trigger immune processes. Some authors do not even include the validation of the specificity of binding MHC and the peptides that they immunochemically isolated in their publications, whereas at the current stage of the development of immunoprecipitation in general, there is no way to completely get rid of non-specific contamination [151]. Any full-fledged study of an immunopeptidome, to search for immunogenic peptides, in addition to in silico bioinformatic validation of the affinity of peptides to MHC, must necessarily include in vitro confirmation of binding to MHC and verification of the immunogenicity of the peptides.

Many researchers of HLA ligands conduct bioinformatics assessment of the binding of these peptides to HLA molecules to test the belonging of identified peptides to an immunopeptidome (for example, using the NetMHC service) [73,85,89,114]. The highest affinity peptides are considered as the most likely constituents of the immunopeptidome. However, despite the constant improvement of in silico methods for predicting affinity, such results still require experimental confirmation. There are several technologies for verifying peptide binding to HLA I: surface plasmon resonance [152], gel filtration [153], and flow cytometry [154]. Marcilla and co-authors analyzed peptides presented by HLA I (allele HLA-B*27) and monkey MHC I (allele Mamu-B*08) [155]. They selected MHC alleles associated with the course of human and simian immunodeficiency. Using a competition-based peptide binding assay employing flow cytometry, the authors identified and compared peptide-binding motifs of different alleles using synthetic peptides. In the competition-based peptide binding assay, cell lines stably expressing MHC I on their surface are subjected to acid treatment to eliminate peptides naturally presented on the MHC. Then, the lines are incubated with the analyzed peptides and control fluorescently labeled peptides; β2 microglobulin is also added since after treatment with acid it could dissociate, destabilizing the overall structure of MHC I. Binding affinity of the target peptide is calculated using flow cytometry through the degree of displacement of labeled control peptide.

To test the identified MHC I ligands on the ability to induce an immune response, the enzyme-linked immunospot (ELISpot) method based on the registration of cytokine signals is used [156]. Immunogenic peptides in MHC I mainly interact with CD8+ T lymphocytes. After interacting with the antigen, killer T cells are activated and release various information molecules: the cytokines. In addition, MHC ligands can interact with various antigen-presenting cells, the activation of which, among other things, can lead to the activation of T lymphocytes. For ELISpot, antibodies specific to certain cytokines are immobilized on the carrier, cells are added (usually PBMCs, including antigen-presenting cells and T-lymphocytes), and peptides, the activity of which is under investigation, are introduced into the system. If peptides have immunogenic properties, then T lymphocytes upon incubation with the peptides release various cytokines into the external environment, which specifically bind to immobilized antibodies. Cells, peptides, and other non-specific components of the system are removed, and the bound cytokines are visualized using antibodies with marker conjugates. Blatnik et al. searched for low-presented immunogenic antigens of the human papillomavirus (HPV), which is the cause of anogenital and oropharyngeal cancer, including cervical cancer [116]. For a few fragments of the E6 and E7 oncotic proteins, 121 antigen candidates were predicted using in silico methods. Of the predicted candidates, 17 peptides were found among the HLA ligandome of HPV-infected cells. These peptides were synthesized, and 11 of them showed immunogenicity in the ELISpot assay on PBMCs from healthy donors.

ELISpot allows one to overcome viral and tumor immunological tolerance. For example, Oh and co-authors investigated the suppression of the presentation of HLA I molecules on the surface of tumor cells as a result of mutations in the mitogen-activated protein kinase (MAPK) cascade proteins [157]. Mutations in proteins participating in the MAPK pathway repress the presentation of antigens by HLA I and, in general, reduce the expression of this protein on the surface of tumor cells, thus hiding the tumor from the body’s immune surveillance [158]. Oh et al. analyzed the effect of inhibitors of mutant proteins ALK (anaplastic lymphoma kinase) and RET (rearranged during transfection) on the HLA I ligandome of malignant cells. They compared the immunopeptidome of tumor cells before and after exposure to inhibitors. New peptides that appeared in the immunopeptidome of cells upon exposure to the inhibitors due to an increase in the expression of HLA I were tested for immunogenicity by the ELISpot method using CD3+ T lymphocytes and CD14+ antigen-presenting macrophages. Among the new peptides, two immunogenic ones were found capable of activating the T-cell response. Thus, they showed the possibility of using ALK and RET inhibitors for T-cell immunotherapy.

A similar method to determine the immunogenicity of identified MHC I ligands is intracellular cytokine staining (ICS) [159]. As in the ELISpot method, T cells are preincubated with the tested peptides, which results in T cell activation (provided that the peptides are immunogenic). The introduction of an inhibitor of protein transport, for example, Brefeldin A, retains newly synthesized cytokines inside the endoplasmic reticulum of the cell. Then, the cells are fixed and permeabilized to allow the penetration of cytokine-specific antibodies. Cells labeled with antibodies specific for cytokines and surface markers are analyzed by flow cytometry. In a study of the LM-MEL-44 melanoma cell line immunopeptidome, Woods and co-authors used ICS to test the immunogenicity of potential tumor-specific peptides, including those found as a result of cell line stimulation with pro-inflammatory IFNγ [160].

It is also possible to establish the immunogenicity of the peptides of interest using MHC I tetramer or multimer assays. For this, primary activation (priming) and expansion of CD8+ T lymphocytes (obtained from healthy PBMCs) are carried out using artificial specific antigen-presenting cells. Then, potentially activated cytotoxic T lymphocytes (CTL) are incubated with fluorescently labeled MHC I tetramers or multimers, which are loaded with the peptide of interest. If priming and expansion were successful, then CTL on the surface will have enough TCR, specific to the antigen under study, which will lead to the interaction with the peptide in tetramers/multimers and cells staining [161]. According to this approach, Schuster et al. have demonstrated the immunogenicity of identified epithelial ovarian cancer derived peptides [95].

## 5. Future Perspectives

Over the past 20–25 years, research in the field of immunopeptidomics has made significant progress, both in methodological terms and in the volume and completeness of the data obtained. The accumulated experience made it possible to improve the immunotherapeutic approach to various oncological diseases [162]. Immunopeptidome analysis has become one of the essential directions of working with adaptive immunity. However, the application of various methodological approaches to this analysis has led to the results that not only cannot be compared with each other but also combined into large associative studies. In the largest to date, to our knowledge, the immunopeptidome study analyzed 10 biosamples of postoperative material and 142 blood plasma samples taken from patients with glioblastoma [89]. Therefore, without a single unified protocol for working with an immunopeptidome, the scientific community faces the difficulty of comparing and combining data and gaining even greater knowledge about the immunopeptidome.

To unite the efforts of the community of immunopeptidome researchers under the auspices of the Human Proteome Organization (HUPO), the Human Immunopeptidome Project (HIPP) consortium was created [163]. The main stated objective of the HUPO-HIPP is to map the entire repertoire of HLA ligands and to make immunopeptidome analysis accessible to any researcher. In particular, this project seeks to conduct association studies of the immunopeptidome in a consortium of large centers or even countries, which could provide a global qualitative and comprehensive analysis of various disease-associated HLA alleles. The idea of such large-scale studies has led Vizcaíno et al. to the concept of an immunopeptidome-wide association study (IWAS): combining the capabilities of many scientific centers to identify correlations between the components of an immunopeptidome and certain human diseases based on studies of large groups of people [164].

We assume that further optimization of research in the field of immunopeptidome will be the automation of experiments to reduce time costs and increase reproducibility. High-throughput protocols for the isolation of immunopeptidome are already beginning to appear, which can allow the robotization of this process [93]. Similar to the massive parallel nucleic acid sequencing, similar strategies for parallel peptide sequencing are already being developed [165], which will allow the analysis of low-abundance samples and mapping of rare amino acid variants.

## Figures and Tables

**Table 1:** Selected examples of association studies that show the broad possibilities of using human leukocyte antigen (HLA) genotyping to predict predisposition to certain diseases; the details of the HLA nomenclature can be found at http://hla.alleles.org/nomenclature/naming.html [22].

Disease	Associated Alleles of the HLA I and II Genes	Study
Parkinson’s disease (PD)	HLA-B*07:02, HLA-C*07:02, HLA-DRB5*01, HLA-DRB1*15:01, HLA-DQA1*01:02, HLA-DQB1*06:02 (positively associated with PD risk) HLA-C*03:04, HLA-DRB1*04:04, HLA-DQA1*03:01 (negatively associated with PD risk)	[9,11]
Birdshot chorioretinopathy	HLA-A*29:02 (>95% of cases carry the HLA-A*29 allele; odds ratio (OR) = 157.5, *p*-Value = 6.6E-74)	[10]
Systemic sclerosis (SSc)	HLA-DRB1*15∶02, HLA-DRB1*16∶02 (major SSc risk allele subtypes) HLA-DRB1*01:01, HLA-DRB1*04:06 (strong SSc-protective)	[12]
Psoriasis	HLA-B*08, HLA-C*06:02, HLA-B*27, HLA-B*38, HLA-B*39 (positively associated with Psoriasis risk)	[13,18]
SARS	HLA-B*46:01 (positively associated with SARS risk, *p*-Value = 0.0279)	[23]
Allergic rhinitis	HLA-B*27 (positively associated with disease risk)	[20]
Lung cancer	HLA-B*08:01, HLA-DQB1*06 (positively associated with lung cancer risk for Europeans) HLA-DQB1*0401, HLA-DRB1*0701 (positively associated with lung cancer risk for Asians)	[21]
Ankylosing spondylitis	HLA-B*27 (positively associated with disease risk)	[24,25]
Behçet’s disease	HLA-B*51 (positively associated with disease risk)	[26,27]
Tuberculosis	rs557011 and rs9271378 (located between HLA-DQA1 and HLA-DRB1) and a missense variant encoding p.Ala210Thr in HLA-DQA1 positively associated with tuberculosis risk	[28]
Crohn’s disease	HLA-C*01 (significant associations with Crohn’s Disease)	[17]
Type-1 diabetes (T1D)	HLA-B*39:06 (positively associated with T1D) HLA-B*38 (protective for T1D) Heterozygous HLA-DQ2/8 (DQA1*05:01-DQB1*02:01/DQA1*03:01-DQB1*03:02) has the highest risk in T1D. Heterozygous HLA-DQ6/8 (DQA1*02:01-DQB1*06:02/ DQA1*03:01-DQB1*03:02) is protective against T1D.	[29,30,31]
Immunoglobulin A deficiency (IgAD)	HLA-DQB1*02, HLA-DRB1*03 and HLA-DRB1*07 (strong IgAD risk factors) HLA-DRB1*15 (protection from IgAD)	[32]
Autoimmune polyglandular syndrome (APS) type 2	HLA-DRB1*03, HLA-DRB1*04, HLA-DQA1*03, HLA-DQB1*02 (positively associated with APS type 2)	[33]
